# What We Know about Sting-Related Deaths? Human Fatalities Caused by Hornet, Wasp and Bee Stings in Europe (1994–2016)

**DOI:** 10.3390/biology11020282

**Published:** 2022-02-11

**Authors:** Xesús Feás, Carmen Vidal, Susana Remesar

**Affiliations:** 1 Academy of Veterinary Sciences of Galicia, 15707 Santiago de Compostela, Spain; 2 Fundación Instituto de Investigación Sanitaria de Santiago de Compostela (IDIS), Hospital Clínico, 15706 Santiago de Compostela, Spain; 3 Servizo de Alergoloxía, Área Sanitaria de Santiago de Compostela e Barbanza, Hospital de Conxo, 15706 Santiago de Compostela, Spain; carmen.vidal.pan@sergas.es or; 4 Área de Medicina, Departamento de Psiquiatría, Radioloxía, Saúde Pública, Enfermaría e Medicina, Facultade de Medicina e Odontoloxía, Universidade de Santiago de Compostela, 15782 Santiago de Compostela, Spain; 5 Animal Health Research: Galicia (INVESAGA), Department of Animal Pathology, Faculty of Veterinary Science, University of Santiago de Compostela, 27002 Lugo, Spain; susana.remesar@usc.es

**Keywords:** Hymenoptera, insect, bee, wasp, hornet, epidemiology, fatalities, venomous animals, public health

## Abstract

**Simple Summary:**

Information about fatalities due to stinging insects is scarce. Hymenopteran-related deaths (*n* = 1691) in 32 European countries based on official registers over a 23-year period (1994–2016) are described. Male adults (25–64 years) were the most common group to be fatally injured and almost half of the fatalities were recorded at “unspecified places”. Fatalities per million inhabitants per year ranged from 0 to 2.24 with an average of 0.26. Geographic, environmental, and ecological factors influence the frequency of stings, and its subsequent reaction. It is necessary to produce and interpret knowledge using diverse sources and in an interdisciplinary way. As part of the One Health philosophy, people and hornets, wasps and bees, as well as the environment that they share are closely connected.

**Abstract:**

Epidemiology of Hymenopteran-related deaths in Europe, based on official registers from WHO Mortality Database (Cause Code of Death: X23), are presented. Over a 23-year period (1994–2016), a total of 1691 fatalities were recorded, mostly occurring in Western (42.8%) and Eastern (31.9%) Europe. The victims tended to concentrate in: Germany (*n* = 327; 1998–2015), France (*n* = 211; 2000–2014) and Romania (*n* = 149; 1999–2016). The majority of deaths occurred in males (78.1%) between 25–64 years (66.7%), and in an “unspecified place” (44.2%). The highest X23MR (mortality rate) were recorded in countries from Eastern Europe (0.35) followed by Western (0.28), Northern (0.23) and Southern Europe (0.2). The countries with the highest and lowest mean X23MR were Estonia (0.61), Austria (0.6) and Slovenia (0.55); and Ireland (0.05), United Kingdom (0.06) and the Netherlands (0.06), respectively. The X23 gender ratio (X23GR; male/female) of mortality varied from a minimum of 1.4 for Norway to a maximum of 20 for Slovenia. Country-by-country data show that the incidence of insect-sting mortality is low and more epidemiological data at the regional level is needed to improve our understanding of this incidence. With the expansion of non-native Hymenopteran species across Europe, allergists should be aware that their community’s exposures are continually changing

## 1. Introduction

Anaphylaxis is an acute, life-threatening reaction that occurs shortly after contact with a trigger. It is classically defined as the most severe form of an allergic reaction, but it could also be induced by non-IgE-mediated pathways through the activation of mast cells and basophils through different mechanisms [1]. Common triggers of anaphylaxis include foods, drugs, venoms from insects, general anaesthetics, radiocontrast agents, and latex, among others, and their prevalence varies from region to region being different for each patient group [2].

The medically important groups of the Hymenoptera order in Europe are those of the genus *Apis* and *Bombus* (belonging to the family Apidae), and *Vespula*, *Dolichovespula*, *Vespa* and *Polistes* (family Vespidae) [3]. Social Apidae, Vespinae and Polistinae possess a large and comprehensive array of venom components [4]. Hymenoptera venoms are composed of a mixture of biologically active proteins and peptides, some of them common to different species such as phospholipases, hyaluronidase, phosphatase, α-glucosidase, serotonin, histamine, dopamine, noradrenaline, and adrenaline. Other proteins such as melittin, apamin, and the mast cell degranulating peptide are exclusive to bees, while mastoparan and bradykinin are only found in wasps [5,6,7,8,9,10]. As a rule, Hymenoptera species are not especially predisposed to attack humans; however, social hornets, wasps, and bees have developed a protective response to whatever risk the colony faces through stings.

With each sting, Hymenopterans inject a small amount of venom that can cause reactions of varying intensity: (i) normal local reactions (NLR), (ii) large local reactions (LLR), (iii) systemic anaphylactic reactions (SAR), (iv) systemic toxic reactions (STR) and (v) unusual reactions (UR) [11]. The local reaction is limited to the area of the sting and is usually small in size, with redness, swelling and intense pain. If a person has a reaction area of the sting of greater than 10 cm, it may mean that he or she has been sensitized, but, as a rule, it does not need any special action. SAR reactions are those that cause symptoms beyond the point of the sting and can range from skin lesions to respiratory problems, digestive symptoms or anaphylaxis. The prevalence of SAR in Europe due to Hymenoptera stings ranged between 0.3 and 7.5% in adults and is slightly lower in children (0.15–3.4%) [12]. STR occur due to multiple Hymenoptera stings because of the well-known toxic properties of their venoms. The symptomatology of UR differs from typical allergic reactions, and, in some cases, follow-up is required over many months [13].

Some authors indicated that 94.5% of humans are stung by *Hymenoptera* insects at some point in their lifetime [14]. Although most of these stings are not reported, general practitioners attend to a large number of patients with Hymenoptera stings, and a few of them require rapid assistant at emergency departments. Thus, in USA, it was estimated that 220,000 annual visits are made to the emergency department and nearly 60 deaths occur per year due to Hymenopteran stings [15]. In addition, an analysis of 4000 cases of anaphylaxis from Germany, Austria and Switzerland, show insect venom (*n* = 2074; 50.1%) as a common trigger of anaphylaxis [2]. For these reasons, researchers highlight that an effective and affordable treatment for anaphylaxis caused by these arthropods is critical.

Hymenopteran stings have recently become a worldwide public health concern [16,17]. However, this health problem can be underestimated despite the number of cases presented. Realistic mortality epidemiological data are still lacking in some countries [18]. Information on the incidence of fatalities due Hymenoptera stings is crucial for an assessment of the problem, as well as to enhance medical assistance for patients and creating public policies aimed at decreasing the incidence of these events [19,20]. More than half of the people with fatal sting reactions had no preceding anaphylactic episodes [21,22].

According to chapter XX of the International Classification of Diseases and Related Health Problems 10th Revision (ICD-10) fatalities due to hornet, wasp and bee stings (including yellow jackets) are coded as X23 [23]. The worldwide incidence of insect-sting mortality ranged from 0.03 to 0.48 fatalities per 1,000,000 inhabitants per year, which is low but not negligible [21].

Information about the fatalities due to stinging insects is scarce. There is a need to improve the epidemiology of these deaths in order to get more accurate, informative and contemporary figures. Therefore, to fill this gap, the aim of the present study was to document and characterize the European deaths caused by Hymenoptera stings during a 23-year period (1994–2016).

## 2. Materials and Methods

**Figure 1:**
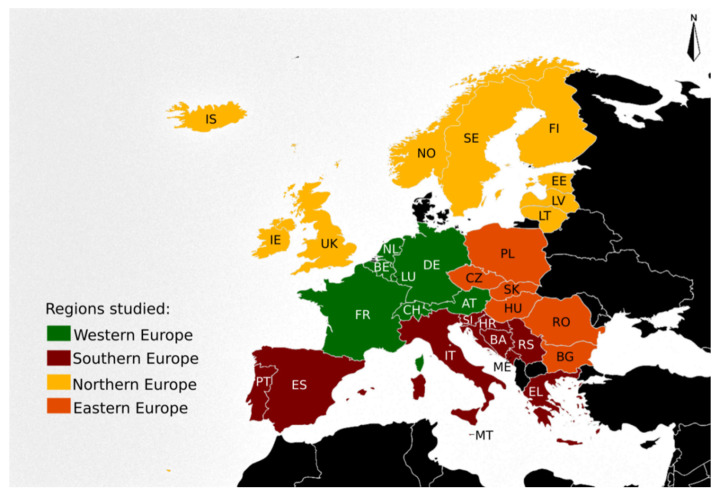
Map of Europe comprising the countries (*n* = 32) included in the study with the four regions assignment: Eastern Europe (in orange): (*n* = 6), (numerical code 151): Bulgaria (BG), Czech Republic (CZ), Hungary (HU), Poland (PL), Romania (RO) and Slovakia (SK). Northern Europe (in green): (*n* = 9), (numerical code 154): Estonia (EE), Finland (FI), Iceland (IS), Ireland (IE), Latvia (LV), Lithuania (LT), Norway (NO), Sweden (SE) and United Kingdom (UK). Southern Europe (in red): (*n* = 10), (numerical code 039): Bosnia and Herzegovina (BA), Croatia (HR), Greece (EL), Italy (IT), Malta (MT), Montenegro (ME), Portugal (PT), Serbia (RS), Slovenia (SI) and Spain (ES). Western Europe (in green): (*n* = 7), (numerical code 155): Austria (AT), Belgium (BE), France (FR), Germany (DE), Luxembourg (LU), Netherlands (NL), and Switzerland (CH). Countries are labelled by their ISO 3166-1 alpha-2 codes [24]. Countries were grouped in four regions, based on the “Standard Country or Area Codes for Statistical Use” (M49) [25], from United Nations geographic scheme for the continent of Europe (numerical code 150), created by the United Nations Statistics Division (UNSD).

The number of deaths related to hornet, wasp and bee stings (including yellow jackets) were obtained from the World Health Organisation (WHO) Mortality Database (MDB). Using X23 as diagnosis code, the MDB returned a file comprising individual data for all X23-related fatalities registered between 1994 and 2016, with both years included. For each observation, the following particular variables were obtained:

− Country in which the fatality happened (*n* = 32) grouped into four regions (Eastern, Northern, Southern and Western Europe) (Figure 1).

− Year of occurrence, from 1994 to 2016.
− X23 code extension to four-digit. Comprising nine categories relative to the place of occurrence: X23.0 (home), X23.1 (residential institution), X23.2 (school, other institution and public administrative area), X23.3 (sports and athletics area), X23.4 (street and highway), X23.5 (trade and service area), X23.6 (industrial and construction area, X23.7 (farm), X23.8 (other specified places) and X23.9 (unspecified place). Supplementary Table S1 lists the included and excluded places in each category.
− Gender, males (♂X23) and females (♀X23).
− Age categories: children (0–14 years), youth (15–24 years), adults (25–64) and seniors (65 years and older).

For each country’s set of data, calculations included: (a) mean, median and mode (central tendency of X23 deaths) and (b) range, variance and standard deviation (variability of X23 deaths). 

The X23 gender ratio (X23GR) of mortality, was obtained by dividing ♂X23, by ♀X23:

X23GR = ♂X23/♀X23(1)

The X23 gender differential (X23GD) in mortality is the absolute difference between ♂X23 and ♀X23:

X23GD = ♂X23 − ♀X23(2)

The X23 mortality rates (X23MR) were on the basis of the yearly number of X23 deaths and the population size in each country:

X23MR = X23deaths/census(3)

The X23MR were expressed in terms of annual rates (i.e., per year) and per 1,000,000 inhabitants. The X23GR, X23GD and X23MR were obtained both for countries and regions, as well as for each year and for the total years of the studied period. Countries censuses were obtained from the European Statistical System (ESS), produced by the statistical office of the European Union (EU) (Eurostat) in partnership with National Statistical Institutes and other national authorities in the EU Member States. The software, Gimp, was used to elaborate a map showing the spatial distribution of the average Hymenopteran sting-related deaths per 1,000,000 inhabitants (X23MR).

## 3. Results

### 3.1. Countries and Study Period

The countries for which data are available, as well as the year period to which they correspond, are presented in Supplementary Table S2. Information was retrievable for a total of 32 countries and in the whole study period there was an annual variation in the data available and provided by each country:

<10 years (*n* = 8): BA, BG, EL, IE, LV, LT, LU and ME.

≥10 years and <20 years (*n* = 15): AT, BE, HR, EE, FR, DE, IT, MT, PL, PT, RO, RS, SI, ES and UK.

≥20 years (*n* = 9): CZ, FI, HU, IS, NL, NO, SK, SE and CH.

### 3.2. Hymenopteran Sting-Related Deaths

During 1994–2016, a total of 1691 deaths were officially registered in Europe with code X23. Fatalities were confirmed in 28 countries, with Germany (*n* = 327, 19.3%), France (*n* = 211, 12.5%) and Romania (*n* = 149, 8.8%) reporting the highest numbers (Table 1). A total of eleven countries (BG, FR, DE, EL, IT, PL, RO, RS, ES, CH and UK) report deaths every year. FR, DE, EL, PL and RO, had the minimum number of such deaths. At least one sting-related death occurred yearly in the following six countries: BG, IT, RS, ES, CH and UK. In the rest of the countries (*n* = 17), deaths due to stings of Hymenoptera insects are not always present. The maximum values of the number of annual deaths for the different countries, varied, ranking as: DE (32), FR (23), RO (16), CZ (14), HU (13); and AT, IT and FL (12). The range of deaths varies from country to country, at it is interesting to highlight the following: DE (range = 26); CZ, FR and RO (range = 14); HU (range = 13); AT (range = 12) and IT (range = 11).

### 3.3. Specific Locations where Hymenopteran Sting-Related Deaths Occurred

The X23 code extension to four-digit is fully available for most of the countries, with a total of 18 reporting case details. Conversely, three countries (FI, LV and SI) report all deaths without the four-digit extension. In seven countries (BE, BG, EE, LT, RS, SK and SE) both the X23 code with and without an extension to four-digit are used (Supplementary Table S3). All countries register the highest number of deaths with the code X23.9 (unspecified place), except for Hungary, and Austria, where the code X23.0 (home) harbours the highest number of Hymenopteran sting-related death events. In general, the pattern observed for most countries, with a few exceptions is X23.9 > X23.0 > X23.8 >X23.4 > X23.7; with no recorded deaths labelled as X23.5 (trade and service area). Very little used codes are: (i) 1 death coded as X23.3 (sports and athletics area) and 1 death coded as X23.6 (industrial and construction area), both having place in Romania; and (ii) seven deaths coded as X23.2 (school, other institution and public administrative area) in Belgium (1), Czechia (1), Hungary (3), Poland (1) and Spain (1); and 8 deaths coded as X23.1 (residential institution) in Austria (1), Belgium (1), Czechia (3), Norway (1) and Poland (2).

### 3.4. Age Distribution of Hymenopteran Sting-Related Deaths

Adults (25–64 years) were the most common age group to be fatally injured by Hymenopteran stings. Lithuania is the only exception, with an equal rate of adults and seniors (65 years and older) Hymenopteran sting-related deaths, a total of three (Supplementary Table S4). Only adult deaths were found in the following two countries: Ireland and Luxembourg. Children (0–14 years) fatalities were recorded in six countries: FR (2), RO (2), AT (1), HR (1), PT (1), and SK (1). A total of 22 victims in the youth group (15–24 years) were recorded in 12 countries with the following distribution: RO (6), AT (3), HU (2) and CH (2), and CZ, FR, DE, IT, LT, PL, RS and SI with only one death.

### 3.5. Hymenopteran Sting-Related Deaths by Gender

The majority of deaths (*n* = 1691) in Europe occurred in males (Supplementary Table S5). In fact, there are four countries with only male deaths, specifically, Greece and Portugal with nine male victims each; and Ireland and Luxembourg with two and one male victims, respectively. The X23 gender ratio (X23GR) of mortality varied from a min value of 1.4 for Norway to a maximum value of 20 for Slovenia, with a range of 18.6 for the whole dataset of countries. Obtained values for the calculated X23 gender differential (X23GD) varied between countries, being over 100 in Germany, France and Romania, with values of 157, 113 and 111, respectively.

### 3.6. Hymenopteran Sting-Related Deaths Features by Region: Eastern, Northern, Southern and Western Europe

Fatalities mostly occurred in Western (42.8%), Eastern (31.9%), Southern (15.1%) and Northern Europe (10.1%). Both X23GR and X23GD varied for the four regions, as follows: X23GD [Western Europe (366), Eastern Europe (310), Southern Europe (190) and Northern Europe (83)] and X23GR [Southern Europe (6.8), Eastern Europe (3.7), Western Europe (3.0) and Northern Europe (2.9)] (Table 2). Results show that eight and twenty-one fatalities were recorded in children (0–14 years) and the youth group (15–24 years), respectively. Adults (25–64 years) and seniors (65 years and older) were the most common age groups to be fatally injured with a total of 1128 victims (66.7%) and 520 victims (30.8%), respectively (Table 2). Fatal stings occurred mainly at home (29.4%) and at other specified places (11.2%), followed by on the street and highway (3.2%) and at farms (1.5%). However, 44.2% of the fatalities, a total of 748, are recorded at an “unspecified place”. The ranked order of the place of ocurrence was: X23.9 (unspecified place) > X23.0 (home) > X23.8 (other specified places) for Europe and Northern Europe; X23.0 (home) > X23.9 (unspecified place) > X23.8 (other specified places) for Eastern Europe; and X23.9 (unspecified place) > X23.8 (other specified places) > X23.0 (home) for Southern Europe.

### 3.7. Hymenopteran Sting-Related Deaths, X23 Mortality Rates (X23MR)

**Figure 2:**
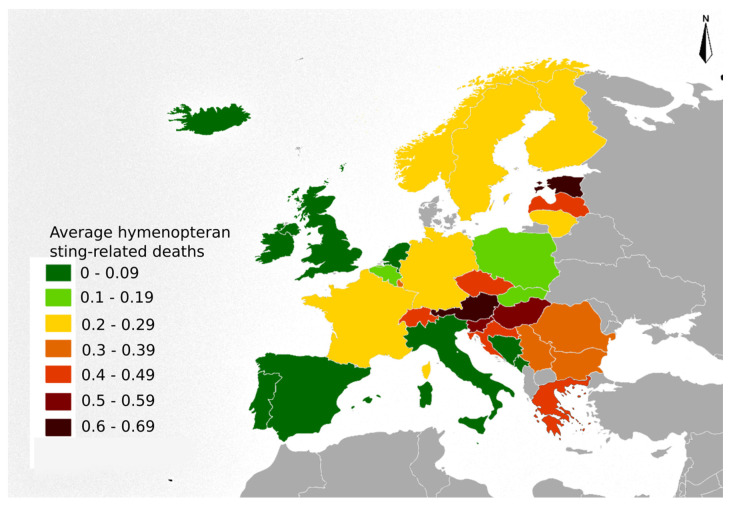
Map of Europe showing the average Hymenopteran sting-related mortality rates (X23MR) during the studied period (1994–2016). The X23MR are expressed in terms of annual rates (i.e., per year) and per 1,000,000 inhabitants, based in the dataset range for each country.

The mortality rates (X23MR) were calculated based on the population size of each country, the obtained results ranged from 0 to 2.24 with an average of 0.26 (Supplementary Table S6). The country with the highest mean X23MR was Estonia (0.61) followed by Austria and Slovenia (0.6 and 0.55, respectively). The countries with the lowest X23MR values were Ireland (0.05), United Kingdom (0.06) and the Netherlands (0.06). The highest rates were recorded in countries from Eastern Europe (X23MR = 0.35) followed by Western (X23MR = 0.28), Northern (X23MR = 0.23) and Southern (X23MR = 0.2) Europe (Figure 2).

## 4. Discussion

There are limited studies of anaphylaxis epidemiology in general, and of insect-venom anaphylaxis in particular, and its impact is probably underestimated and largely unknown. One of the reasons is the difficult coding of the condition under the ICD-10 [26]. The WHO officially included allergic and hypersensitivity conditions as a disorder in the ICD-11 in May 2019 [27,28,29]. The ICD-11 came into effect on January 2022, and the “Injury, poisoning or certain other consequences of external causes” is now chapter XXII, and “External causes of morbidity or mortality” is now chapter XXIII. The term “Contact with hornets, wasps and bees” was replaced to “Unintentionally stung or envenomated by animal” (code PA78). The different insects could be postcoordinated by the following extension codes: XE4D9 Bee, XE6LT Wasp and XE322 Hornet [30]. In adidition, it can be found “Allergic or hypersensitivity reactions to arthropods”, represented by code 4A85.3. This includes both local cutaneous and systemic allergic and hypersensitivity reactions to contact with insects and other arthropods, with the following codes in this section: (a) systemic allergic reaction due to Hymenoptera venom (4A85.30), (b) cutaneous allergic or hypersensitivity reactions to Hymenoptera venom (4A85.31) and (c) cutaneous allergic or hypersensitivity reactions to arthropods (4A85.32).

The present study succeeded in gathering new and recent epidemiological information on Hymenopteran sting-related deaths in Europe. The majority of deaths occurred in adult males (25–64 years) at “unspecified places”. The incidence observed in men may be related to different occupational roles and exposure degrees. This situation has been reported especially for beekeepers, electrical facility field workers, farming activities and forestry workers [31,32,33]. Moreover, mastocytosis, a risk factor for venom allergy, is more common in males than in females [34].

Related to the place of occurrence, almost half of the fatalities were recorded at “unspecified places”. This makes it more difficult to develop prevention initiatives. Increased specificity in the place coding of deaths would help public health professionals target prevention interventions.

Hymenopteran-induced fatalities differ by age. The number of deaths clearly indicates that adult (1128 = 66.7%) and elderly (570 = 30.8%) people are at a higher risk agreeing with previous studies carried out in Costa Rica, South Korea and UK [35,36,37]. Children do not usually shown an anaphylactic reaction after Hymenopteran stings, being the LLR the most common presentation [38,39,40].

Historical available X23MR values for European countries were found at 0.09 in England and Wales, 13 yr period (1959–1971) [41]; 0.2 in Sweden, 10 yr period (1975–1984) [42]; 0.18 in Germany, 5 yr period (1979–1983) [43]; 0.25 in Denmark, 21 yr period (1960–1980) [44]; 0.43 in France, 12 yr period (1981–1991) [45] and 0.45 in Switzerland, 3 yr period (1961–1963) [46]. The raw number of Hymenopteran-related deaths provides, at the country level, medical authorities with quantitative information of interest, but it is less comparable across countries due to large differences in population. Equally important, detailed studies of the incidence of deaths from stings at the regional level within each country are desirable. As evidenced recently, sting-related deaths regional epidemiological data differ considerably from the average country rate [47].

Honeybees and the business of apiculture have economic importance for agricultural production and beehive products. However, beekeepers and those living in proximity to hives are under sting risk [33]. In 2010 the total number of honeybee colonies in the European Union was close to 13.845.070, with a high heterogeneity distribution [48]. Spain, Greece and France have the highest density of colonies (≈10 colony/km^2^); however, the countries located in the extreme north of Europe have the lowest density (1 colony/km^2^ or less). There is not a high presence of beehives in either country with the highest X23MR in Estonia (X23MR = 0.61; 42,000 hives; 1 hives/km^2^; 3.2 hives/100 inhabitants), Austria (X23MR = 0.6; 367,583 hives; 4.4 hives per km^2^ and per 100 inhabitants) and Slovenia (X23MR = 0.55; 48,990 hives; 17.2 hives/km^2^; 7.6 hives/100 inhabitants).

Although wasps are disliked by the general public, there is evidence and reasons to consider the regulatory, provisioning, supporting and cultural ecosystem services value of wasps on par with other insects such as bees [49,50]. Vespid species, populations, and ecosystems shown marked biogeographical differences throughout Europe [51]. It is necessary to point out that, within the past two decades, not-native species have been detected in Europe: (i) the yellow-legged Asian hornet (*Vespa velutina* Lepeletier 1836), found in southwestern France in 2004 [52]; (ii) the black shield hornet (*Vespa bicolor* Fabricius, 1787) found in 2013 in the community of Andalucía in Spain [53] and the (iii) American paper wasp (*Polistes major major* Palisot de Beauvois 1818) found in 2008 in Asturias in northern Spain [54]. In addition, some vespids have been translocated from certain European countries to others, such as (i) the Oriental hornet (*Vespa orientalis* Linnaeus 1771) naturally distributed in the south-eastern Europe, found in eastern Spain (Community of Valencia) in 2012 [55], in southern Spain in Algeciras in 2018 [56], in the Northern part of Bucharest in Romania in 2019 [57], in southern France in 2021 [58], and in the central-northern of Italy in the city of Florence in 2021 [59]; and (ii) *Vespula* and *Dolichovespula* species were confirmed in Iceland, Shetland, Orkney and the Faroe Islands [60]. In the entrance areas, the species have maintained stable populations, but they differ in their ability to propagate, highlighting the fact that the *Vespa velutina* is a highly invasive alien species.

With the expansion of the above mentioned non-native Hymenopteran species across Europe, allergists should be aware that their community’s exposures are continually changing. In this way, the invasive *Vespa velutina* was identified as responsible for three-quarters of the Hymenoptera anaphylaxis patients reported during the last years in north-western Spain [61,62]. Invasive species have a major impact on human health, biodiversity and economics [63,64,65,66]. One Health philosophy has been adopted by the European Academy of Allergy and Clinical Immunology (EAACI). Such recognition led to the establishment of an independent Working Group (WG) within the EAACI solely dedicated to One Health in 2021 [67]. Because of the highly interdisciplinary nature of this WG, it is desirable that EAACI members work together with colleagues from a variety of fields. Veterinarians should play an important role in this work, based on their expertise, working closely with the beekeepers and recognizing the insects involved in the attacks. Lastly, descriptions of the stings have important value in this study since it has been reported that the identification of these insects performed by general population and allergy specialists is poor [40,68,69].

## 5. Conclusions

The most obvious shortcoming of the present investigation is that the hymenoptera species involved were not individually identified, with data provided by ICD-10 code. Knowledge about the presence of different stinging insects in Europe is important for diagnostic and therapeutic purposes. Our study has the advantage of using a large country-based data set, which allow us to analyze time trends and age/gender distributions of hymenoptera sting-related deaths. However, epidemiological data at the regional level is needed to improve our understanding of Hymenoptera-sting incidence.

The frequency of stings and allergic reactions depends on different variables such as geographic, environmental, and ecological factors. When dealing with complicated challenges such as the health risks caused by native and not-native species of bee and wasp stings, it is necessary to produce, assemble and interpret information and knowledge using diverse sources and in an interdisciplinary way. As part of the One Health philosophy, people and animals as well as the environment that they share are closely connected. With the expansion of not-native Hymenopteran species across Europe, allergists should be aware that their community’s exposures are continually changing and include these insects as causes of sting-related allergic reactions.

## Figures and Tables

**Table 1:** Descriptive statistics of fatalities due to hornet, wasp and bee stings by country (1994–2016).

Country ^1^	Size	Sum	Minimum	Maximum	Range	Mode	Mean	Median	Standard Deviation	Variance
AT	15	73	0	12	12	5	4.87	5	3.27	10.7
BE	18	27	0	5	5	1	1.5	1	1.42	2.03
BG	9	26	1	6	5	2	2.89	2	1.62	2.61
HR	19	35	0	6	6	1	1.84	1	1.71	2.92
CZ	23	111	0	14	14	4	4.83	4	3.04	9.24
EE	17	14	0	3	3	0	0.82	1	1.01	1.03
FI	20	25	0	4	4	0	1.25	1	1.25	1.57
FR	15	211	9	23	14	14	14.07	14	4.15	17.21
DE	18	327	6	32	26	18	18.17	18	6.97	48.62
EL	2	9	4	5	1	4 & 5	4.5	4.5	0.71	0.5
HU	21	112	0	13	13	4	5.33	4	3.54	12.53
IE	8	2	0	1	1	0	0.25	0	0.46	0.21
IT	13	69	1	12	11	5 & 3	5.31	5	3.4	11.56
LV	9	9	0	2	2	2	1	1	1	1
LT	7	7	0	4	4	1 & 0	1	1	1.41	2
LU	7	1	0	1	1	0	0.14	0	0.38	0.14
NL	21	20	0	5	5	0	0.95	1	1.2	1.45
NO	20	19	0	3	3	1	0.95	1	0.76	0.58
PL	17	125	3	12	9	7 & 8 & 9	7.35	8	2.67	7.12
PT	13	9	0	4	4	0	0.69	0	1.32	1.73
RO	18	149	2	16	14	10	8.28	9	3.44	11.86
RS	18	53	1	6	5	3	2.94	3	1.43	2.06
SK	21	17	0	2	2	0	0.81	1	0.81	0.66
SI	19	21	0	4	4	0	1.11	1	1.37	1.88
ES	17	60	1	8	7	4 & 3	3.53	3	1.81	3.26
SE	20	44	0	6	6	1	2.2	2	1.61	2.59
CH	21	65	1	8	7	4	3.1	3	1.61	2.59
UK	15	51	1	7	6	2	3.4	4	1.84	3.4

^1^ There are not registered deaths due to hornet, wasp and bee stings in: Iceland (1996–2016), Malta (1995–2004), Montenegro (1999–2004) and Bosnia and Herzegovina (2011).

**Table 2:** Absolute and relative frequency of the deaths due to hornet, wasp and bee stings in Europe and by European region: counts, X23 gender ratio of mortality (X23GR), X23 gender differential in mortality (X23GD), by age distribution and by place of occurrence.

Items	Items	Eastern Europe ^1^ 1994–2016	Northern Europe ^2^ 1996–2016	Southern Europe ^3^ 1995–2015	Western Europe ^4^ 1995–2016	Europe 1994–2016
Deaths	Total	540	171	256	724	1691
	Men	425 (78.7%)	127 (74.3%)	223 (87.1%)	545 (75.3%)	1320 (78.1%)
	Woman	115 (21.3%)	44 (25.7%)	33 (12.9%)	179 (24.7%)	371 (21.9%)
Ratio	X23GR	3.7	2.9	6.8	3	3.6
	X23GD	310	83	190	366	949
Age	≤14 years	3 (0.6%)	-	2 (0.8%)	3 (0.4%)	8 (0.5%)
	15–24 years	10 (1.9%)	1 (0.6%)	3 (1.2%)	7 (1%)	21 (1.2%)
	25–64 years	418 (77.4%)	101 (59.1%)	169 (66%)	440 (60.8%)	1128 (66.7%)
	≥65 years	109 (20.2%)	55 (32.2%)	82 (32%)	274 (37.8%)	520 (30.8%)
Place	Not reported	33 (6.1%)	52 (30.4%)	73 (28.5%)	1 (0.1%)	159 (9.4%)
	X23 code extension to four-digit reported ^5^	507 (93.9%)	119 (69.6%)	183 (71.5%)	723 (99.9%)	1532 (90.6%)
	X23.0 (home)	241 (44.6%)	35 (20.5%)	19 (7.4%)	202 (27.9%)	497 (29.4%)
	X23.1 (residential institution)	5 (0.9%)	1 (0.6%)	-	2 (0.3%)	8 (0.5%)
	X23.2 (school, other institution and public administrative area)	5 (0.9%)	-	1 (0.4%)	1 (0.1%)	7 (0.4%)
	X23.3 (sports and athletics area)	1 (0.2%)	-	-	-	1 (0.1%)
	X23.4 (street and highway)	17 (3.1%)	1 (0.6%)	6 (2.3%)	30 (4.1%)	54 (3.2%)
	X23.5 (trade and service area)	-	-	-	-	-
	X23.6 (industrial and construction area)	1 (0.2%)	-	-	-	1 (0.1%)
	X23.7 (farm)	18 (3.3%)	1 (0.6%)	6 (2.3%)	1 (0.1%)	26 (1.5%)
	X23.8 (other specified place)	48 (8.9%)	9 (5.3%)	21 (8.2%)	112 (15.5%)	190 (11.2%)
	X23.9 (unspecified place)	171 (31.7%)	72 (42.1%)	130 (50.8%)	375 (51.8%)	748 (44.2%)

^1^ Eastern Europe (*n* = 6): BG, CZ, HU, PL, RO and SK. ^2^ Northern Europe (*n* = 9): EE, FI, IS, IE, LV, LT, NO, SE and UK. ^3^ Southern Europe (*n* = 10): BA, HR, EL, IT, MT, ME, PT, RS, SI and ES. ^4^ Western Europe (*n* = 7): AT, BE, FR, DE, LU, NL, and CH. ^5^ Supplementary Table S1 lists the included and excluded places in each category.

## Data Availability

Not applicable.

## Supplementary Materials

The following are available online at https://www.mdpi.com/article/10.3390/biology11020282/s1, Supplementary Table S1: Place of occurrence code. The following categories are provided to be used as separate variables in addition to ICD categories W00-Y34 to identify the place of occurrence of the external cause where relevant. Supplementary Table S2: List of all European countries (*n* = 32) analyzed in the present study with their country code, dataset range and number of datasets. Supplementary Table S3: Absolute and relative frequency of the deaths due to hornet, wasp and bee stings by place of occurrence. Supplementary Table S4: Absolute and relative frequency of the deaths due to hornet, wasp and bee stings by age. Supplementary Table S5: Absolute and relative frequency of the deaths due to hornet, wasp and bee stings by gender; and the X23 gender ratio (X23GR) of mortality and the X23 gender differential (X23GD) in mortality. Supplementary Table S6: Hymenopteran sting-related mortality rates (X23MR) during the studied period (1994–2016) calculated on the basis of the yearly population size of each country as reported on the census, as of 1 January of each year. The X23MR are expressed per year and per 1,000,000 inhabitants.
