# Supplementary material for: What We Know about Sting-Related Deaths? Human Fatalities Caused by Hornet, Wasp and Bee Stings in Europe (1994–2016)
Source: Biology (Basel). 2022 Feb 11;11(2):282. doi: 10.3390/biology11020282 (PMC8869362; doi:10.3390/biology11020282)
Supplement: Supplementary file 1 [file biology-11-00282-s001.zip › Supplementary Table S1.pdf]

Supplementary Table S1. Place of occurrence code. The following categories are provided to to be used as separate variables in addition to ICD categories W00-Y34 to identify the place of occurrence of the external cause where relevant.

Code	Place	Included	Excluded*
0	Home.	Apartment; Boarding-house; Caravan [trailer] park, residential; Farmhouse; Home premises; House (residential); Noninstitutional place of residence; Private: driveway to home, garage, garden to home and yard to home; and Swimming-pool in private house or garden.	(i) Abandoned or derelict house[8]; (ii) home under construction but not yet occupied[6] and (iii) institutional place of residence[1].
1	Residential institution.	Children's home; Dormitory; Home for the sick; Hospice; Military camp; Nursing home; Old people's home; Orphanage; Pensioner's home; Prison; and Reform school.	
2	School, other institution and public administrative area.	Building (including adjacent grounds) used by the general public or by a particular group of the public such as: assembly hall, campus, church, cinema, clubhouse, college, court-house, dancehall, day nursery, gallery, hospital, institute for higher education, kindergarten, library, movie-house, museum, music-hall, opera-house, post office, public hall, school (private)(public)(state), theatre, university and youth centre.	(i) Building under construction[6]; (ii) residential institution [1] and (iii) sports and athletics area [3].
3	Sports and athletics area.	Baseball field; Basketball-court; Cricket ground; Football field; Golf-course; Gymnasium; Hockey field; Riding-school; Skating-rink; Squash-court; Stadium; Swimming-pool, public; and Tennis-court.	Swimming-pool or tennis-court in private home or garden[0].
4	Street and highway.	Freeway; Motorway; Pavement; Road and Sidewalk.	
5	Trade and service area.	Airport; Bank; Café; Casino; Garage (commercial); Gas station; Hotel; Market; Office building; Petrol station; Radio or television station; Restaurant; Service station; Shop (commercial); Shopping mall; Station (bus)(railway); Store; Supermarket and Warehouse.	Garage in private home[0].
6	Industrial and construction area.	Building [any] under construction; Dockyard; Dry dock; Factory: building and premises; Gasworks; Industrial yard; Mine; Oil rig and other offshore installations; Pit (coal)(gravel)(sand); Power-station (coal)(nuclear)(oil); Shipyard; Tunnel under construction and Workshop.	
7	Farm.	Farm: (i) buildings, (ii) land under cultivation and (iii) ranch.	Farmhouse and home premises of farm[0].
8	Other specified places.	Beach; Campsite; Canal; Caravan site NOS; Derelict house; Desert; Dock NOS; Forest; Harbour; Hill; Lake; Marsh; Military training ground; Mountain; Park (amusement) (public); Parking-lot and parking-place; Pond or pool; Prairie; Public place NOS; Railway line; River; Sea; Seashore; Stream; Swamp; Water reservoir and Zoo.	
9	Unspecified place		

* The superscript number corresponds to the category in which those places should be included.
