# Supplementary material for: What We Know about Sting-Related Deaths? Human Fatalities Caused by Hornet, Wasp and Bee Stings in Europe (1994–2016)
Source: Biology (Basel). 2022 Feb 11;11(2):282. doi: 10.3390/biology11020282 (PMC8869362; doi:10.3390/biology11020282)
Supplement: Supplementary file 1 [file biology-11-00282-s001.zip › Supplementary Table S2.pdf]

Supplementary Table S2. List of all European countries (n=32) analyzed in the present study with their country code, dataset range and number of datasets.

Country	Country code[1]	Dataset range (year), first–last	Number of datasets
Austria	AT	2002-2016	15
Belgium	BE	1998-2015	18
Bosnia and Herzegovina	BA	2011	1
Bulgaria	BG	2005-2013	9
Croatia	HR	1995-2013	19
Czechia	CZ	1994-2016	23
Estonia	EE	1997-2013	17
Finland	FI	1996-2015	20
France	FR	2000-2014	15
Germany	DE	1998-2015	18
Greece	EL	2014-2015	2
Hungary	HU	1996-2016	21
Iceland	IS	1996-2016	21
Ireland	IE	2007-2013	8
Italy	IT	2003-2015	13
Latvia	LV	1996-2004	9
Lithuania	LT	1998-2004	7
Luxembourg	LU	1998-2004	7
Malta	MT	1995-2004	10
Montenegro	ME	1999-2004	5
Netherlands	NL	1996-2016	21
Norway	NO	1996-2015	20
Poland	PL	1999-2015	17
Portugal	PT	2002-2014	13
Romania	RO	1999-2016	18
Serbia	RS	1998-2015	18
Slovakia	SK	1994-2014	21
Slovenia	SI	1997-2015	19
Spain	ES	1999-2015	17
Sweden	SE	1997-2016	20
Switzerland	CH	1995-2015	21
United Kingdom	UK	2001-2015	15

[1] Based on the ISO 3166-1 alpha-2 code [24].
