# Supplementary material for: What We Know about Sting-Related Deaths? Human Fatalities Caused by Hornet, Wasp and Bee Stings in Europe (1994–2016)
Source: Biology (Basel). 2022 Feb 11;11(2):282. doi: 10.3390/biology11020282 (PMC8869362; doi:10.3390/biology11020282)
Supplement: Supplementary file 1 [file biology-11-00282-s001.zip › Supplementary Table S3.pdf]

Supplementary Table S3. Absolute and relative frequency of the deaths due to hornet, wasp and bee stings by place of occurrence.

Country	X23[1]	X23.four digit	Home (X23.0)	Residential institution (X23.1)	Public area (X23.2)	Sporting area (X23.3)	Street and highway (X23.4)	Trade and service area (X23.5)	Industrial area (X23.6)	Farm (X23.7)	Others (X23.8)	Unspecified place (X23.9)
AT	-	73 (100%)	11 (15.1%)	1 (1.4%)	-	-	-	-	-	-	39 (53.4%)	22 (30.1%)
BE	1 (3.7%)	26 (96.3%)	6 (22.2%)	1 (3.7%)	1 (3.7%)	-	3 (11.1%)	-	-	-	2 (7.4%)	13 (48.1%)
BA	-	-	-	-	-	-	-	-	-	-	-	-
BG	20 (76.9%)	6 (23.1%)	2 (7.7%)	-	-	-	-	-		2 (7.7%)	2 (7.7%)	-
HR	-	35 (100%)	13 (37.1%)	-	-	-	-	-	-	-	9 (25.7%)	13 (37.1%)
CZ	-	111 (100%)	39 (35.1%)	3 (2.7%)	1 (0.9%)	-	1 (0.9%)	-	-	2 (1.8%)	-	65 (58.6%)
EE	10 (71.4%)	4 (28.6%)	-	-	-	-	-	-	-	-	-	4 (28.6%)
FI	25 (100%)	-	-	-	-	-	-	-	-	-	-	-
FR	-	211 (100%)	88 (41.7%)	-	-	-	25 (11.8%)	-	-	1 (0.5%)	5 (2.4%)	92 (43.6%)
DE	-	327 (100%)	96 (29.4%)	-	-	-	2 (0.6%)	-	-	-	65 (19.9%)	164 (50.2%)
EL	-	9 (100%)	1 (11.1%)	-	-	-	-	-	-	-	-	8 (88.9%)
HU	-	112 (100%)	46 (41.1%)	-	3 (2.7%)	-	14 (12.5%)	-	-	7 (6.3%)	24 (21.4%)	18 (16.1%)
IS	-	-	-	-	-	-	-	-	-	-	-	-
IE	-	2 (100%)	-	-	-	-	-	-	-	-	-	2 (100%)
IT	-	69 (100%)	3 (4.3%)	-	-	-	3 (4.3%)	-	-	1 (1.4%)	8 (11.6%)	54 (78.3%)

LV	9 (100%)	-	-	-	-	-	-	-	-	-	-	-
LT	1 (14.3%)	6 (85.7%)	5 (71.4%)	-	-	-	-	-	-	-	-	1 (14.3%)
LU		1 (100%)	-	-	-	-	-	-	-	-	-	1 (100%)
MT	-	-	-	-	-	-	-	-	-	-	-	-
ME	-	-	-	-	-	-	-	-	-	-	-	-
NL	-	20 (100%)	1 (5%)	-	-	-	-	-	-	-	-	19 (95%)
NO	-	19 (100%)	2 (10.5%)	1 (5.3%)	-	-	-	-	-	-	1 (5.3%)	15 (78.9%)
PL	-	125 (100%)	28 (22.4%)	2 (1.6%)	1 (0.8%)	-	-	-	-	5 (4%)	17 (13.6%)	72 (57.6%)
PT	-	9 (100%)	-	-	-	-	-	-	-	-	-	9 (100%)
RO	-	149 (100%)	125 (83.9%)	-	-	1 (0.7%)	2 (1.3%)	-	1 (0.7%)	2 (1.3%)	3 (2%)	15 (10.1%)
RS	52 (98.1%)	1 (1.9%)	-	-	-	-	-	-	-	1 (1.9%)	-	-
SK	13 (76.5%)	4 (23.5%)	1 (5.9%)	-	-	-	-	-	-	-	2 (11.8%)	1 (5.9%)
SI	21 (100%)	-	-	-	-	-	-	-	-	-	-	-
ES	-	60 (100%)	2 (3.3%)	-	1 (1.7%)	-	3 (5%)	-	-	4 (6.7%)	4 (6.7%)	46 (76.7%)
SE	7 (15.9%)	37 (84.1%)	-	-	-	-	-	-	-	-	-	37 (84.1%)
CH	-	65 (100%)	-	-	-	-	-	-	-	-	1 (1.5%)	64 (98.5%)
UK	-	51 (100%)	28 (54.9%)	-	-	-	1 (2%)	-	-	1 (2%)	8 (15.7%)	13 (25.5%)

[1] **Supplementary Table S1** lists the included and excluded places in each category: X23.0, X23.1, X23.2, X23.3, X23.4, X23.5, X23.6, X23.7, X23.8 and X23.9.
