# Supplementary material for: What We Know about Sting-Related Deaths? Human Fatalities Caused by Hornet, Wasp and Bee Stings in Europe (1994–2016)
Source: Biology (Basel). 2022 Feb 11;11(2):282. doi: 10.3390/biology11020282 (PMC8869362; doi:10.3390/biology11020282)
Supplement: Supplementary file 1 [file biology-11-00282-s001.zip › Supplementary Table S4.pdf]

Supplementary Table S4. Absolute and relative frequency of the deaths due to hornet, wasp and bee stings by age.

Country	Children (≤ 14 years)	Youth (15-24 years)	Adults (25-64 years)	Seniors (≥ 65 years)
Austria	1 (1.4%)	3 (4.1%)	43 (58.9%)	26 (35.6%)
Belgium	0	0	19 (70.4%)	8 (29.6%)
Bosnia and Herzegovina	0	0	0	0
Bulgaria	0	0	20 (76.9%)	6 (23.1%)
Croatia	1 (2.9%)	0	29 (82.9%)	5 (14.3%)
Czechia	0	1 (0.9%)	81 (73%)	29 (26.1%)
Estonia	0	0	12 (85.7%)	2 (14.3%)
Finland	0	0	18 (72%)	7 (28%)
France	2 (0.9%)	1 (0.5%)	110 (52.1%)	98 (46.4%)
Germany	0	1 (0.3%)	215 (65.7%)	111 (33.9%)
Greece	0	0	5 (55.6%)	4 (44.4%)
Hungary	0	2 (1.8%)	86 (76.8%)	24 (21.4%)
Iceland	0	0	0	0
Ireland	0	0	2 (100%)	0
Italy	0	1 (1.4%)	42 (60.9%)	26 (37.7%)
Latvia	0	0	8 (88.9%)	1 (11.1%)
Lithuania	0	1 (14.3%)	3 (42.9%)	3 (42.9%)
Luxembourg	0	0	1 (100%)	0
Malta	0	0	0	0
Montenegro	0	0	0	0
Netherlands	0	0	14 (70%)	6 (30%)
Norway	0	0	10 (52.6%)	9 (47.4%)
Poland	0	1 (0.8%)	95 (76%)	29 (23.2%)
Portugal	1 (11.1%)	0	6 (66.7%)	2 (22.2%)
Romania	2 (1.3%)	6 (4%)	123 (82.6%)	18 (12.1%)
Serbia	0	1 (1.9%)	41 (77.4%)	11 (20.8%)
Slovakia	1 (5.9%)	0	13 (76.5%)	3 (17.6%)
Slovenia	0	1 (4.8%)	15 (71.4%)	5 (23.8%)
Spain	0	0	31 (51.7%)	29 (48.3%)
Sweden	0	0	25 (56.8%)	19 (43.2%)
Switzerland	0	2 (3.1%)	38 (58.5%)	25 (38.5%)
United Kingdom	0	0	35 (68.6%)	16 (31.4%)
