# Supplementary material for: What We Know about Sting-Related Deaths? Human Fatalities Caused by Hornet, Wasp and Bee Stings in Europe (1994–2016)
Source: Biology (Basel). 2022 Feb 11;11(2):282. doi: 10.3390/biology11020282 (PMC8869362; doi:10.3390/biology11020282)
Supplement: Supplementary file 1 [file biology-11-00282-s001.zip › Supplementary Table S5.pdf]

Supplementary Table S5. Absolute and relative frequency of the deaths due to hornet, wasp and bee stings by gender; and the X23 gender ratio (X23GR) of mortality and the X23 gender differential (X23GD) in mortality.

Country	Men	Woman	% Men	% Woman	X23GR[1]	X23GD[2]
AT	54	19	74	26	2.8	35
BE	20	7	74.1	25.9	2.9	13
BA	-	-	-	-	-	-
BG	24	2	92.3	7.7	12	22
HR	28	7	80	20	4	21
CZ	75	36	67.6	32.4	2.1	39
EE	11	3	78.6	21.4	3.7	8
FI	20	5	80	20	4	15
FR	162	49	76.8	23.2	3.3	113
DE	242	85	74	26	2.8	157
EL	9	0	100	0	nc	9
HU	82	30	73.2	26.8	2.7	52
IS	-	-	-	-	-	-
IE	2	0	100	0	nc	2
IT	59	10	85.5	14.5	5.9	49
LV	8	1	88.9	11.1	8	7
LT	6	1	85.7	14.3	6	5
LU	1	0	100	0	nc	1
MT	-	-	-	-	-	-
ME	-	-	-	-	-	-
NL	18	2	90	10	9	16
NO	11	8	57.9	42.1	1.4	3
PL	98	27	78.4	21.6	3.6	71
PT	9	0	100	0	nc	9
RO	130	19	87.2	12.8	6.8	111
RS	45	8	84.9	15.1	5.6	37
SK	16	1	94.1	5.9	16	15
SI	20	1	95.2	4.8	20	19
ES	53	7	88.3	11.7	7.6	46
SE	33	11	75	25	3	22
CH	48	17	73.8	26.2	2.8	31
UK	36	15	70.6	29.4	2.4	21

[1] The X23 gender ratio (X23GR) of mortality, was obtained by dividing the number of male who died from a X23 cause (♂X23), by the number of female who died from a X23 cause (♀X23).

[2] The X23 gender differential (X23GD) in mortality is the absolute difference of the male (♂X23) and female (♀X23) who died from a X23 cause.
