# Supplementary material for: What We Know about Sting-Related Deaths? Human Fatalities Caused by Hornet, Wasp and Bee Stings in Europe (1994–2016)
Source: Biology (Basel). 2022 Feb 11;11(2):282. doi: 10.3390/biology11020282 (PMC8869362; doi:10.3390/biology11020282)
Supplement: Supplementary file 1 [file biology-11-00282-s001.zip › Supplementary Table S6.pdf]

**Supplementary Table S6.** Hymenopteran sting-related mortality rates (X23MR) during the studied period (1994-2016) calculated on the basis of the yearly population size of each country as reported on the census, as of 1 January of each year. The X23MR are expressed per year and per 1,000,000 inhabitants.

| | 1994 | 1995 | 1996 | 1997 | 1998 | 1999 | 2000 | 2001 | 2002 | 2003 | 2004 | 2005 | 2006 | 2007 | 2008 | 2009 | 2010 | 2011 | 2012 | 2013 | 2014 | 2015 | 2016 | Min | Max | Standard deviation | Average |
|---|---|---|---|---|---|---|---|---|---|---|---|---|---|---|---|---|---|---|---|---|---|---|---|---|---|---|---|
| **AT** | | | | | | | | | 0 | 0.25 | 0.38 | 0.63 | 1.51 | 0.88 | 0.62 | 0.87 | 0.62 | 1.23 | 0.37 | 0.12 | 0.61 | 0.24 | 0.72 | 0 | 1.51 | 0.41 | 0.6 |
| **BE** | | | | | 0.1 | 0 | 0.1 | 0.1 | 0.19 | 0.19 | 0.39 | 0.1 | 0 | 0.1 | 0 | 0.49 | 0 | 0.19 | 0 | 0.29 | 0.19 | 0.19 | | 0 | 0.49 | 0.14 | 0.15 |
| **BA** | | | | | | | | | | | | | | | | | | 0 | | | | | | 0 | 0 | 0 | 0 |
| **BG** | | | | | | | | | | | | 0.13 | 0.26 | 0.4 | 0.67 | 0.27 | 0.27 | 0.27 | 0.41 | 0.82 | | | | 0.13 | 0.82 | 0.22 | 0.39 |
| **HR** | | 0.43 | 0.22 | 0.66 | 0 | 0 | 0.22 | 0.23 | 0.23 | 0.7 | 0.23 | 0 | 0.46 | 0.23 | 0.46 | 0.93 | 0.46 | 0 | 1.4 | 1.17 | | | | 0 | 1.4 | 0.4 | 0.42 |
| **CZ** | 0.39 | 0.39 | 0.68 | 0.68 | 0.87 | 0.29 | 0.39 | 0.59 | 0 | 1.37 | 0.29 | 0.29 | 0.39 | 0.2 | 0.58 | 0.86 | 0.19 | 0.67 | 0.29 | 0.48 | 0.19 | 0.28 | 0.38 | 0 | 1.37 | 0.3 | 0.47 |
| **EE** | | | | 0 | 0 | 0 | 0.71 | 0 | 2.17 | 0.73 | 0.73 | 0.74 | 0 | 0 | 2.24 | 0.75 | 0.75 | 0 | 1.51 | 0 | | | | 0 | 2.24 | 0.75 | 0.61 |
| **FI** | | | 0 | 0.39 | 0 | 0.58 | 0 | 0.39 | 0.19 | 0.38 | 0.19 | 0 | 0.19 | 0 | 0 | 0 | 0.37 | 0.37 | 0.37 | 0.55 | 0.73 | 0 | | 0 | 0.73 | 0.23 | 0.24 |
| **FR** | | | | | | | 0.17 | 0.18 | 0.16 | 0.23 | 0.37 | 0.32 | 0.16 | 0.19 | 0.22 | 0.22 | 0.23 | 0.22 | 0.23 | 0.14 | 0.3 | | | 0.14 | 0.37 | 0.06 | 0.22 |
| **DE** | | | | | 0.22 | 0.26 | 0.24 | 0.17 | 0.13 | 0.36 | 0.39 | 0.22 | 0.35 | 0.12 | 0.15 | 0.22 | 0.2 | 0.25 | 0.17 | 0.2 | 0.07 | 0.27 | | 0.07 | 0.39 | 0.08 | 0.22 |
| **EL** | | | | | | | | | | | | | | | | | | | | | 0.37 | 0.46 | | 0.37 | 0.46 | 0.07 | 0.41 |
| **HU** | | | 0.19 | 0.39 | 0.39 | 0.2 | 0.78 | 0.1 | 0.39 | 0.39 | 0.3 | 0.2 | 0.3 | 0.5 | 0.8 | 0.4 | 0 | 0.8 | 1.11 | 1.31 | 1.01 | 0.91 | 0.71 | 0 | 1.31 | 0.36 | 0.53 |
| **IS** | | | 0 | 0 | 0 | 0 | 0 | 0 | 0 | 0 | 0 | 0 | 0 | 0 | 0 | 0 | 0 | 0 | 0 | 0 | 0 | 0 | 0 | 0 | 0 | 0 | 0 |
| **IE** | | | | | | | | | | | | | 0 | 0 | 0.22 | 0 | 0.22 | 0 | 0 | 0 | 0 | | | 0 | 0.22 | 0.1 | 0.05 |
| **IT** | | | | | | | | | | 0.09 | 0.09 | 0.03 | 0.09 | 0.02 | 0.05 | 0.15 | 0.19 | 0.2 | 0.1 | 0.05 | 0.07 | 0.05 | | 0.02 | 0.2 | 0.06 | 0.09 |
| **LV** | | | 0.81 | 0.82 | 0 | 0.42 | 0 | 0.85 | 0.86 | 0 | 0 | | | | | | | | | | | | | 0 | 0.86 | 0.42 | 0.42 |

| | | | | | | | | | | | | | | | | | | | | | | | | | | | |
|---|---|---|---|---|---|---|---|---|---|---|---|---|---|---|---|---|---|---|---|---|---|---|---|---|---|---|---|
| **LT** | | | | | 0.28 | 1.13 | 0 | 0 | 0 | 0.29 | 0.29 | | | | | | | | | | | | | 0 | 1.13 | 0.4 | 0.29 |
| **LU** | | | | | 0 | 0 | 0 | 0 | 0 | 0 | 2.2 | | | | | | | | | | | | | 0 | 2.2 | 0.83 | 0.31 |
| **MT** | | 0 | 0 | 0 | 0 | 0 | 0 | 0 | 0 | 0 | 0 | | | | | | | | | | | | | 0 | 0 | 0 | 0 |
| **ME** | | | | | | | 0 | 0 | 0 | 0 | 0 | | | | | | | | | | | | | 0 | 0 | 0 | 0 |
| **NL** | | | 0.32 | 0.06 | 0.06 | 0 | 0.13 | 0 | 0.06 | 0 | 0.06 | 0 | 0.06 | 0.12 | 0.06 | 0.06 | 0 | 0 | 0 | 0.12 | 0 | 0 | 0.12 | 0 | 0.32 | 0.08 | 0.06 |
| **NO** | | | 0.69 | 0.46 | 0.23 | 0.22 | 0.22 | 0.22 | 0.22 | 0.22 | 0.22 | 0 | 0 | 0.21 | 0 | 0.21 | 0.21 | 0 | 0.4 | 0 | 0.2 | 0.19 | | 0 | 0.69 | 0.17 | 0.21 |
| **PL** | | | | | | 0.18 | 0.21 | 0.08 | 0.18 | 0.21 | 0.24 | 0.1 | 0.26 | 0.24 | 0.24 | 0.16 | 0.18 | 0.21 | 0.32 | 0.11 | 0.08 | 0.29 | | 0.08 | 0.32 | 0.07 | 0.19 |
| **PT** | | | | | | | | | 0.1 | 0 | 0 | 0 | 0 | 0.28 | 0.09 | 0 | 0 | 0 | 0 | 0 | 0.38 | | | 0 | 0.38 | 0.13 | 0.07 |
| **RO** | | | | | | 0.22 | 0.49 | 0.4 | 0.46 | 0.55 | 0.46 | 0.47 | 0.38 | 0.14 | 0.39 | 0.2 | 0.44 | 0.1 | 0.5 | 0.45 | 0.25 | 0.81 | 0.4 | 0.1 | 0.81 | 0.17 | 0.4 |
| **RS** | | | | | 0.13 | 0.26 | 0.4 | 0.4 | 0.27 | 0.67 | 0.54 | 0.13 | 0.4 | 0.27 | 0.41 | 0.68 | 0.55 | 0.83 | 0.42 | 0.28 | 0.42 | 0.14 | | 0.13 | 0.83 | 0.2 | 0.4 |
| **SK** | 0.19 | 0.19 | 0.37 | 0.19 | 0 | 0.19 | 0.19 | 0 | 0 | 0.37 | 0.19 | 0 | 0 | 0 | 0.19 | 0.37 | 0 | 0 | 0.37 | 0 | 0.37 | | | 0 | 0.37 | 0.15 | 0.15 |
| **SI** | | | | 0.5 | 0 | 0 | 0.5 | 0 | 0.5 | 1.5 | 0 | 0 | 0.5 | 1.49 | 0 | 1.48 | 0 | 1.46 | 0 | 0.49 | 0 | 1.94 | | 0 | 1.94 | 0.67 | 0.55 |
| **ES** | | | | | | 0.1 | 0.07 | 0.1 | 0.12 | 0.12 | 0.05 | 0.09 | 0.07 | 0.02 | 0.02 | 0.13 | 0.04 | 0.09 | 0.06 | 0.04 | 0.17 | 0.06 | | 0.02 | 0.17 | 0.04 | 0.08 |
| **SE** | | | | 0.68 | 0.11 | 0.11 | 0.11 | 0 | 0.34 | 0.22 | 0.22 | 0 | 0.11 | 0.33 | 0.33 | 0.11 | 0.11 | 0.32 | 0.11 | 0.52 | 0.31 | 0.31 | 0.41 | 0 | 0.68 | 0.17 | 0.24 |
| **CH** | | 0.57 | 0.28 | 0.42 | 1.13 | 0.28 | 0.14 | 0.42 | 0.28 | 0.55 | 0.54 | 0.27 | 0.54 | 0.53 | 0.13 | 0.39 | 0.26 | 0.64 | 0.38 | 0.5 | 0.12 | 0.36 | | 0.12 | 1.13 | 0.22 | 0.42 |
| **UK** | | | | | | | | 0.02 | 0.03 | 0.1 | 0.12 | 0.07 | 0.07 | 0.07 | 0.03 | 0.08 | 0.08 | 0.03 | 0.02 | 0.03 | 0.06 | 0.03 | | 0.02 | 0.12 | 0.03 | 0.06 |
